# Clinical characteristics and neuropsychiatric profiles of young-onset dementia: a retrospective cross-sectional study at a tertiary care center in Northern Thailand

**DOI:** 10.3389/fpsyt.2026.1912953

**Published:** 2026-09-03

**Authors:** Sirivipa Jekpoo, Kittithatch Booncharoen, Supisara Prompila, Tinakon Wongpakaran, Huali Wang, Nahathai Wongpakaran

**Affiliations:** 1 Department of Psychiatry, Faculty of Medicine, Chiang Mai University, Chiang Mai, Thailand; 2 Center of Medical Excellence, Faculty of Medicine, Chiang Mai University, Chiang Mai, Thailand; 3 Northern Neuroscience Center, Faculty of Medicine, Chiang Mai University, Chiang Mai, Thailand; 4 Dementia Care and Research Center, Peking University Institute of Mental Health (Sixth Hospital), Beijing, China

**Keywords:** young-onset dementia, Alzheimer’s disease, vascular dementia, frontotemporal dementia, neuropsychiatric symptoms, Thailand

## Abstract

**Background:**

Data on young-onset dementia (YOD) in Thailand remain limited. We aimed to describe clinical presentation, general cognitive performance, neuropsychiatric symptoms, neuroimaging features, and selected laboratory findings across YOD subtypes in a tertiary-care cohort in Northern Thailand.

**Methods:**

We conducted a retrospective cross-sectional study of 295 individuals with YOD (symptom onset < 65 years) treated at Chiang Mai University Hospital (2010–2025). Data extracted included demographics, presenting complaints, general cognitive scores (MoCA/TMSE/MSET-10), neuropsychiatric symptoms (NPI-Q, TGDS), functional status, neuroimaging findings, and laboratory results (HIV, syphilis serology, glycemic indices, and lipid profiles).

**Results:**

Alzheimer’s disease (AD) and vascular dementia (VaD) were the most common subtypes. Memory impairment was the most frequent presenting complaint overall (78.3%) and was more common in AD than VaD (93.4% vs 77.8%), whereas behavioral change was more common in VaD than AD (31.1% vs 13.2%). General cognitive scores did not significantly differ between AD and VaD. Neuropsychiatric symptoms were frequent, with aberrant motor behavior (42.2%) and irritability/lability (35.1%) being the most prevalent domains. Regarding neuroimaging features, a substantial burden of subcortical and basal ganglia lesions co-occurred predominantly within the VaD group. Behavioral variant frontotemporal dementia (bvFTD) accounted for 3.7% of cases; most met the International Behavioral Variant FTD Criteria Consortium (FTDC) criteria for probable bvFTD. All tested participants had non-reactive HIV results, and reactive syphilis screening results were uncommon and were not supported by confirmatory treponemal testing.

**Conclusion:**

Presenting complaints differed between young-onset AD and VaD despite similar general cognitive performance, underscoring the importance of informant history and multimodal assessment for accurate subtyping. Frequent motor-related neuropsychiatric symptoms highlight the clinical relevance of subcortical/basal ganglia lesion burden in this cohort.

## 1 Introduction

Young-onset dementia (YOD) is defined by the onset of symptoms before the age of 65 years (1). A systematic review and meta-analysis reported a global age-standardized prevalence of YOD at 119.0 per 100,000 population, corresponding to an estimated 3.9 million people worldwide (2). YOD imposes substantial psychosocial, financial, and caregiving burdens for people with dementia (PWD) and their families (1). Because symptoms often begin in midlife, many affected individuals are still employed and may be supporting children or aging parents, leading to major disruptions in work, family roles, and household finances (3, 4).

Compared with late-onset dementia, YOD encompasses a broader range of underlying etiologies. Although Alzheimer’s disease (AD), frontotemporal dementia (FTD), and vascular dementia (VaD) are the most common causes (5), YOD frequently involves rarer pathologies, secondary conditions, or atypical presentations. Presentations frequently include behavioral changes, language difficulties, and executive dysfunction rather than isolated memory loss (3), which may contribute to a diagnostic delay that commonly ranges from 3.4 to 4.4 years (5, 6).

Young-onset dementia remains under-recognized in many low- and middle-income countries, where limited access to specialist services and advanced biomarkers may delay diagnosis. In Thailand, recent data from the Collaborative Aging and Dementia Research Society Thailand (CART) registry shows that YOD represents 17% of all dementia cases nationwide. The CART registry identifies the primary causes of YOD as early-onset amnestic Alzheimer’s disease 44%, vascular dementia 16%, and behavioral variant frontotemporal dementia 8% (7). This distribution is consistent with recent data from the Neurological Institute of Thailand in Bangkok, which demonstrated that typical amnestic AD, VaD, and FTD were the most prevalent etiologies (8). However, current literature often lacks systematic reporting on the behavioral and psychological symptoms of dementia (BPSD) within the Thai clinical context. A large cohort study demonstrated that the overall prevalence of most BPSDs is significantly higher in early-onset AD (EOAD) compared to late-onset AD (LOAD), most notably apathy (62.5%), agitation (49.6%), depression (49.4%), and irritability (48.1%) (9). Even in studies where the overall symptom severity was found to be higher in LOAD, depression remained distinctively prominent in EOAD (10). Similarly, in patients with vascular impairments, particularly subcortical vascular cognitive impairment (VCI), the underlying vascular burden and subsequent disruption of subcortical networks play a major role in driving high rates of apathy, depression, and irritability or agitation. Furthermore, when individuals present with mixed dementia, there is a notably higher frequency of hallucinations, sleep disorders, and nighttime behavior disorders (11).

In resource-limited settings where advanced biomarkers are inaccessible, routine laboratory findings may serve as adjunctive tools to support clinical subtype differentiation and guide management. Laboratory investigations represent a clinically important yet underreported component of YOD assessment. A large nationwide cohort study demonstrated that metabolic syndrome—defined by abnormalities in waist circumference, blood pressure, fasting glucose, triglycerides, and HDL cholesterol—was associated with a 24% increased risk of all-cause YOD, a 12% increased risk of AD, and a 21% increased risk of VaD (12). While brain imaging and detailed clinical profiles are generally more critical for definitive subtype classification, screening for these cardiometabolic parameters in younger patients is valuable for identifying manageable comorbidities and understanding the overall vascular burden.

Understanding the demographic, clinical, neuropsychiatric, and investigation profiles of YOD is essential to improve early recognition and management. Therefore, this study aimed to describe the clinical characteristics of YOD in a tertiary-care cohort in Northern Thailand and to compare presenting complaints, cognitive screening performance, neuropsychiatric symptoms, functional status, and laboratory/metabolic profiles between the two most common subtypes (AD and VaD). We also describe neuroimaging patterns in VaD and summarize clinical characteristics of bvFTD in this cohort.

## 2 Materials and methods

### 2.1 Study design and setting

We conducted a retrospective cross-sectional study using medical records from Chiang Mai University Hospital, a university-based tertiary referral center in Northern Thailand. Records of patients diagnosed with major neurocognitive disorder (dementia) were identified using International Classification of Diseases, 10th Revision (ICD-10) codes. The study period spanned January 2010 to June 2026. The study cohort included individuals evaluated at both the outpatient clinics and inpatients who required psychiatric or neurologic consultation within the hospital.

### 2.2 Participants

Patients were eligible if they:

1. Received a clinical diagnosis of major neurocognitive disorder or dementia during the study period.
2. Met criteria for young-onset dementia (YOD), defined as symptom onset before 65 years of age.

We excluded patients with late-onset dementia (symptom onset ≥ 65 years), incomplete records that precluded determination of age at onset or diagnostic classification, and duplicate encounters (only the first eligible visit/diagnosis was retained).

### 2.3 Ethics approval

This study was approved by the Research Ethics Committee, Faculty of Medicine, Chiang Mai University, Chiang Mai, Thailand (Psy-2568-0861).

### 2.4 Data collection and variables

Demographic variables at the time of diagnosis included age, sex, and educational level. Clinical variables included age at symptom onset, time from symptom onset to diagnosis, comorbidities (e.g., hypertension, dyslipidemia, diabetes mellitus, prior stroke/TIA), and presenting complaints. Age at onset and presenting complaints were extracted from clinician documentation and collateral history from caregivers when available.

Cognitive assessments included the Thai version of Montreal Cognitive Assessment (MoCA-T), Thai Mental State Examination (TMSE), and the Mental Status Examination Thai-10 (MSET-10), depending on availability in routine care. During data cleaning, records labeled as “MMSE” in the medical chart were verified to correspond to the MSET-10 instrument and were reclassified as MSET-10 for analysis; therefore, MMSE was not analyzed as a separate instrument. MoCA form (standard MoCA vs MoCA-B) was not consistently documented; therefore, MoCA scores were analyzed as recorded in clinical practice. (Only include this if you indeed cannot distinguish MoCA vs MoCA-B reliably.) Neuropsychiatric symptoms were assessed using the Neuropsychiatric Inventory Questionnaire (NPI-Q) and 15-item Thai Geriatric Depression Scale (TGDS-15). Dementia severity was evaluated using the Global Deterioration Scale (GDS) and Clinical Dementia Rating (CDR).

While routine initial clinical evaluations for dementia at our center comprehensively included standard laboratory investigations, such as thyroid function tests, serum vitamin B12 levels, renal function tests, liver function tests, and a complete blood count (CBC) to screen for reversible causes, the specific laboratory data extracted and analyzed for the purpose of this study focused on syphilis serology (non-treponemal screening with confirmatory treponemal testing when available), anti-HIV testing, fasting blood sugar (FBS), hemoglobin A1c (HbA1c), and lipid profiles.

### 2.5 Diagnostic classification

Dementia subtypes (e.g., Alzheimer’s disease, vascular dementia, frontotemporal dementia) were assigned based on clinical evaluation documented in the medical record and available investigations, including neuroimaging (CT/MRI). For bvFTD, diagnostic stratification followed the International Consensus Criteria (FTDC) (13), as all FTD cases identified within our retrospective cohort during the study period presented solely with the behavioral phenotype, with no cases meeting the diagnostic criteria for language variants. Specifically, diagnoses of Alzheimer’s disease and vascular dementia were defined in accordance with the ICD-10 diagnostic criteria.

### 2.6 Statistical analysis

All statistical analyses were performed using IBM SPSS Statistics, Version 29 (IBM Corp., Armonk, NY, USA). Continuous variables were summarized as mean ± standard deviation (SD) or median (interquartile range [IQR]) depending on distribution, which was assessed using visual inspection and normality testing (e.g., Shapiro–Wilk test). Categorical variables were summarized as frequencies and percentages. The primary inferential analyses compared patients with Alzheimer’s disease (AD) and vascular dementia (VaD), the two most prevalent subtypes in this cohort. Other dementia subtypes were not included in between-group inferential comparisons because of small sample sizes. Between-group comparisons for categorical variables were conducted using chi-square tests (or Fisher’s exact test when expected cell counts were small). Continuous variables were compared using independent-samples t-tests for approximately normally distributed data and Mann–Whitney U tests for non-normally distributed data. All tests were two-sided, and p-values < 0.05 were considered statistically significant. Analyses were conducted using available-case data; therefore, denominators vary across variables, and percentages were calculated using the number of participants with available data for each analysis. To evaluate the missing data mechanism for key variables, we performed Little’s MCAR test and summarized missingness patterns. Because education was missing in a substantial proportion of participants, we conducted sensitivity analyses using multivariable linear regression within each cognitive-test subsample (cognitive score as the dependent variable; predictors included dementia subtype, age, and sex) and repeated these models including education using multiple imputation (m = 5). For comparisons across multiple NPI-Q domains, we controlled multiple testing using the Benjamini–Hochberg false discovery rate (q = 0.05).

## 3 Results

### 3.1 General characteristics of study participants

**Figure 1:**
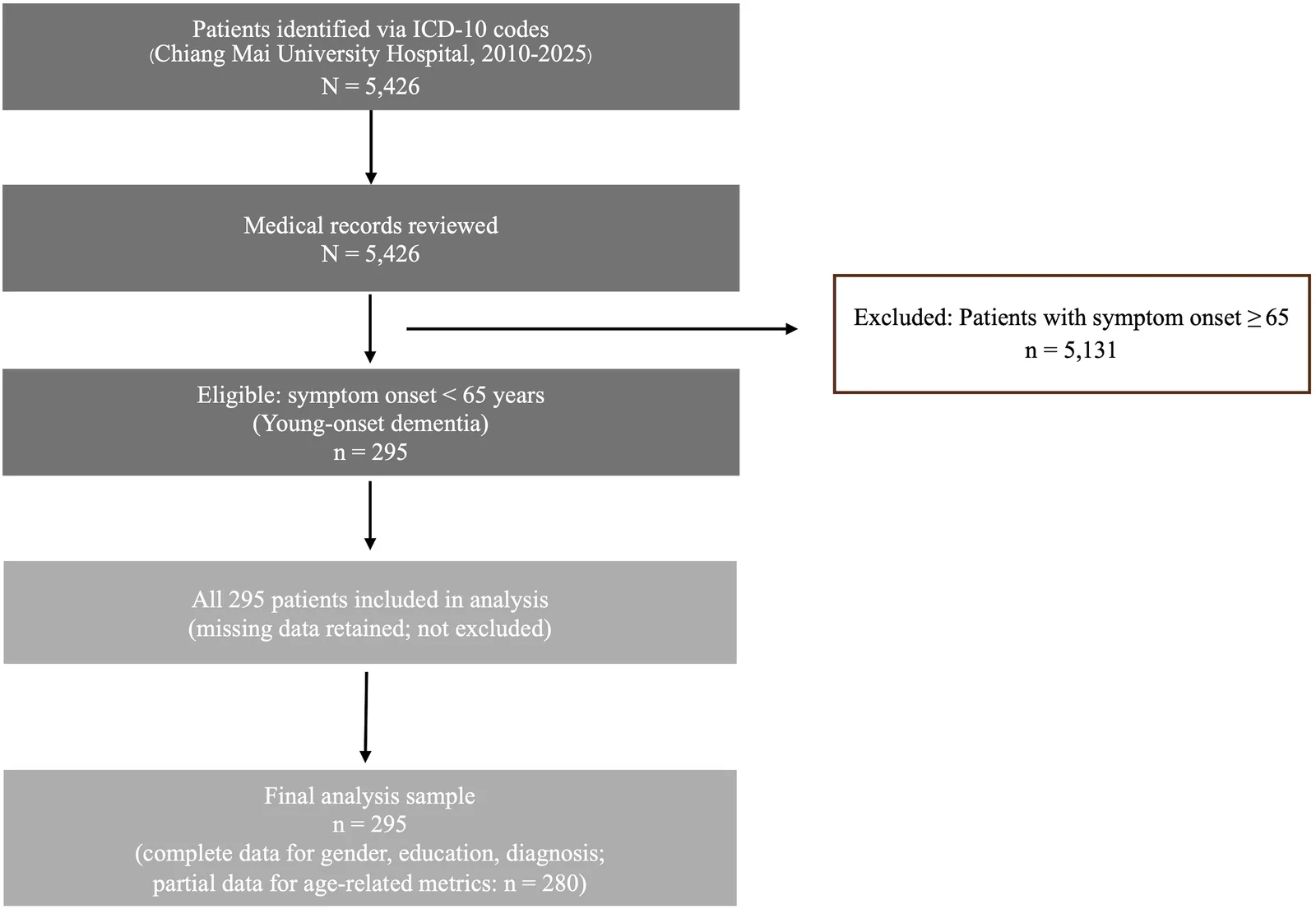
Flowchart of study participant selection. A total of 5,426 cases were initially identified through ICD-10 records over a 15-year period. Following the exclusion of late-onset dementia cases, 295 patients met the criteria for young-onset dementia and were included in the final analysis.

A total of 295 participants with YOD were included in the analysis. The participant selection process is shown in **Figure 1**. The baseline characteristics of patients are presented in **Table 1**. Mean age at diagnosis was 63.83 ± 7.67 years, and mean age at symptom onsets was 58.88 ± 6.19 years. The mean duration from symptom onset to diagnosis was 24.99 ± 31.90 months (n = 280); education data were missing 48.5% of patients. Females comprised 56.6% of the cohort. Hypertension (46.4%) and dyslipidemia (37.6%) were the most common comorbidities. The most common dementia subtypes were Alzheimer’s disease (30.8%) and vascular dementia (30.5%), followed by Parkinson’s disease dementia (5.4%), major neurocognitive disorder due to another medical condition (5.4%), and frontotemporal dementia (3.7%).

**Table 1:** Baseline characteristics of the young-onset dementia.

Characteristics	Total (N = 295)		n (valid)
Demographic data			
Age at diagnosis (years), mean ± SD Age at onset (years), mean ± SD Symptoms onset-to-diagnosis (months), mean ± SD Sex: Female, n (%) Education, n (%) - Below primary - Primary education - Lower secondary education - Upper secondary education - Vocational education - Higher education - No data		63.83 ± 7.67 58.88 ± 6.19 24.99 ± 31.90 167 (56.6) 16 (5.4) 70 (23.7) 13 (4.4) 21 (7.1) 3 (1) 29 (9.8) 143 (48.5)	295 294 280 295 295
Clinical profiles			
Underlying disease, n (%) - Hypertension - Dyslipidemia - Type 2 diabetes mellitus - History of Stroke or TIA Diagnosis, n (%) - AD - VaD - FTD - Mixed dementia - Parkinson’s disease dementia - Dementia with Lewy bodies - Major neurocognitive disorder due to another medical condition - Others		137 (46.4) 111 (37.6) 62 (21) 58 (19.7) 91 (30.8) 90 (30.5) 11 (3.7) 9 (3.1) 16 (5.4) 10 (3.4) 16 (5.4) 52 (17.6)	295 295

Data are presented as mean ± SD or n (%). n (valid) indicates the number of participants with available data for each variable.

SD, standard deviation; AD, Alzheimer’s disease; VaD, vascular dementia; FTD, frontotemporal dementia; TIA, transient ischemic attack.

**Figure 2:**
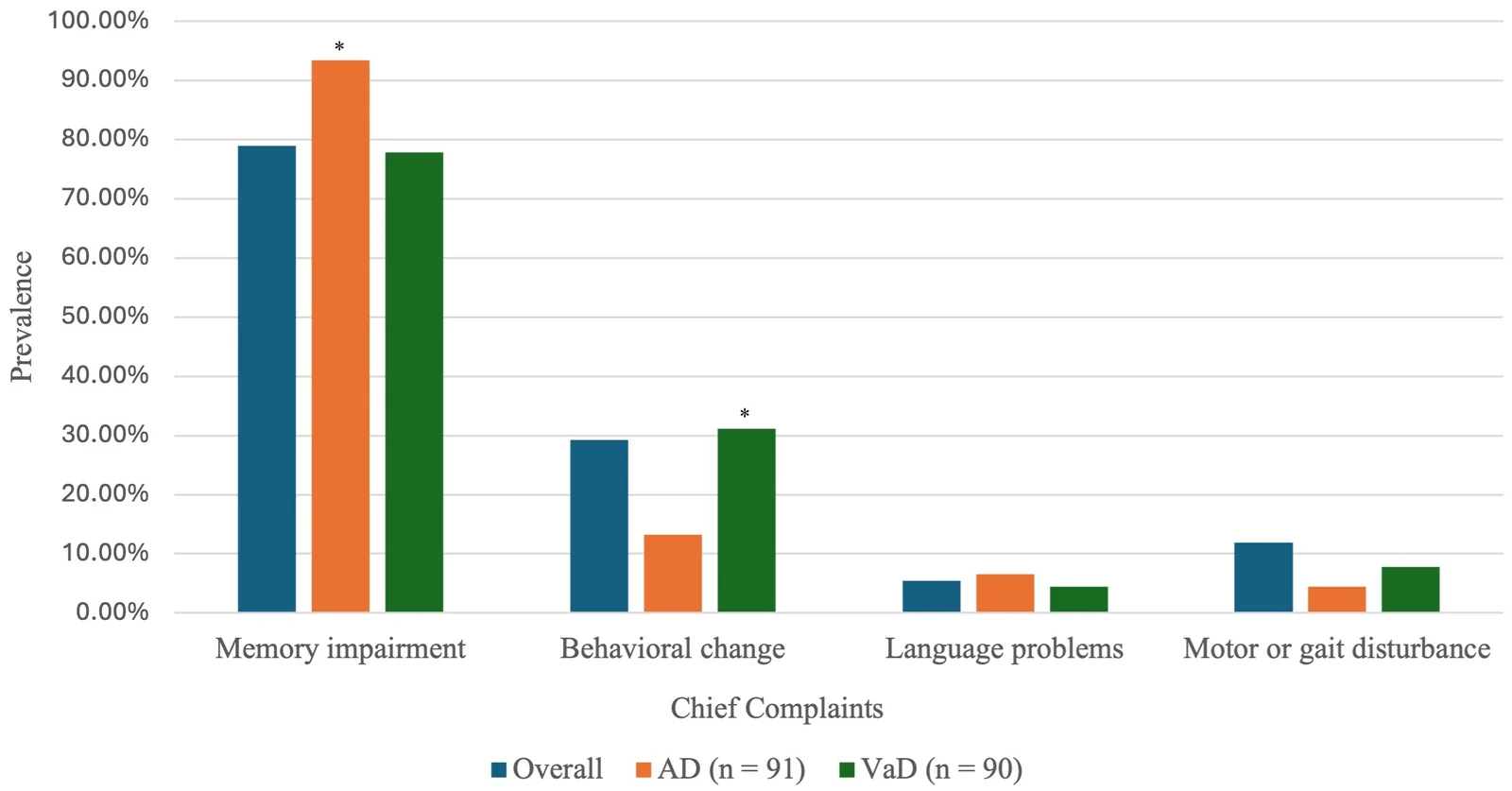
Comparison of chief complaints across the total cohort and dementia subgroups. The bar chart displays the prevalence of primary presenting symptoms for the total sample (N = 295), AD (n = 91), and VaD (n = 90). *p < 0.05 denotes significant differences between AD and VaD groups, calculated using the Likelihood Ratio Chi-square test. AD, Alzheimer’s disease; VaD, vascular dementia.

Overall, the most common presenting complaint was memory impairment (78.3%), followed by behavioral change (29.2%) (**Figure 2**). Presenting complaints were not mutually exclusive. Memory complaints were more frequent in the AD group than the VaD group (93.4% vs 77.8%, p = 0.002), whereas behavioral change was more frequent in VaD than AD (31.1% vs 13.2%, p = 0.003).

### 3.2 Comparison of clinical characteristics and cognitive profiles in young-onset AD and VaD

Cognitive assessment availability varied across instruments (MoCA n = 138; TMSE n = 104; MSET-10 n = 156). Following data verification, records originally labeled as MMSE were identified as MSET-10 and were reclassified accordingly; therefore, MMSE was not analyzed as a separate instrument. Because the “overall” availability includes participants with other etiologies, the AD vs VaD subgroup comparisons were based on smaller instrument-specific samples (MoCA: AD n = 50, VaD n = 44; TMSE: AD n = 35, VaD n = 34; MSET-10: AD n = 50, VaD n = 51) (**Table 2**). Mean MoCA and MSET-10 scores and median TMSE scores did not differ significantly between AD and VaD (MoCA p = 0.395; TMSE p = 0.576; MSET-10 p = 0.099); these comparisons should be interpreted cautiously given substantial missingness and limited power for detecting modest differences. Dementia severity measures were available for 215 patients with CDR and 281 patients with GDS. CDR category distributions differed between AD and VaD (p = 0.017), with a higher proportion of moderate-to-severe impairment (CDR 2–3) in AD (13.4%) than VaD (1.5%). In contrast, GDS severity distributions did not differ between groups (p = 0.658), with both groups predominantly classified as mild-to-moderate dementia (GDS 4–5).

**Table 2:** Cognitive profiles and severity measures in young-onset dementia patients, overall and by dementia subtype (AD and VaD).

Cognitive profiles	Overall*	AD	VaD	p-value†
Cognitive scores				
MoCA score	15.72 ± 6.28 (n = 138)	15.86 ± 6.46 (n = 50)	14.75 ± 6.08 (n = 44)	0.395
MSET-10 score	18.00 ± 5.86 (n = 156)	16.74 ± 6.46 (n = 50)	18.65 ± 4.97 (n = 51)	0.099
TMSE score, median (IQR)	20 (16.5, 23.5) (n = 104)	21 (11, 24) (n = 35)	20 (17, 23) (n = 34)	0.576
Severity scores				
CDR degree, n (%) - Normal - Very mild (0.5) - Mild (1) - Moderate to severe (2-3)	(n = 215) 3 (1) 158 (53.6) 40 (13.6) 14 (4.7)	(n = 67) 1 (1.5) 47 (70.1) 10 (14.9) 9 (13.4)	(n = 68) 1 (1.5) 47 (69.1) 19 (27.9) 1 (1.5)	0.017
GDS, n (%) - Mild cognitive impairment (1-3) - Mild to moderate dementia (4-5) - Severe dementia (6-7)	(n = 281) 70 (24.9) 198 (70.5) 13 (4.4)	(n = 87) 21 (24.1) 62 (71.3) 4 (4.6)	(n = 88) 17 (19.3) 68 (77.3) 3 (3.4)	0.658

Data are presented as n (%) or mean ± SD, except for TMSE score which is presented as median (IQR).

*Percentages and p-values for all columns (Overall, AD, and VaD) are calculated based on valid cases only (the number of patients with available data). The specific number of valid cases (n) for each analysis is provided in parentheses.

**†**p-value compares AD and VaD groups only, using Independent T-test, or Mann-Whitney U test as appropriate.

AD, Alzheimer’s disease; VaD, vascular dementia, MoCA, Montreal Cognitive Assessment; TMSE, Thai Mental State Examination; MSET-10, The Mental Status Examination Thai 10; CDR, Clinical Dementia Rating; GDS, Global Deterioration Scale.

### 3.3 Comparison of neuropsychiatric symptoms in young-onset AD and VaD

**Figure 3:**
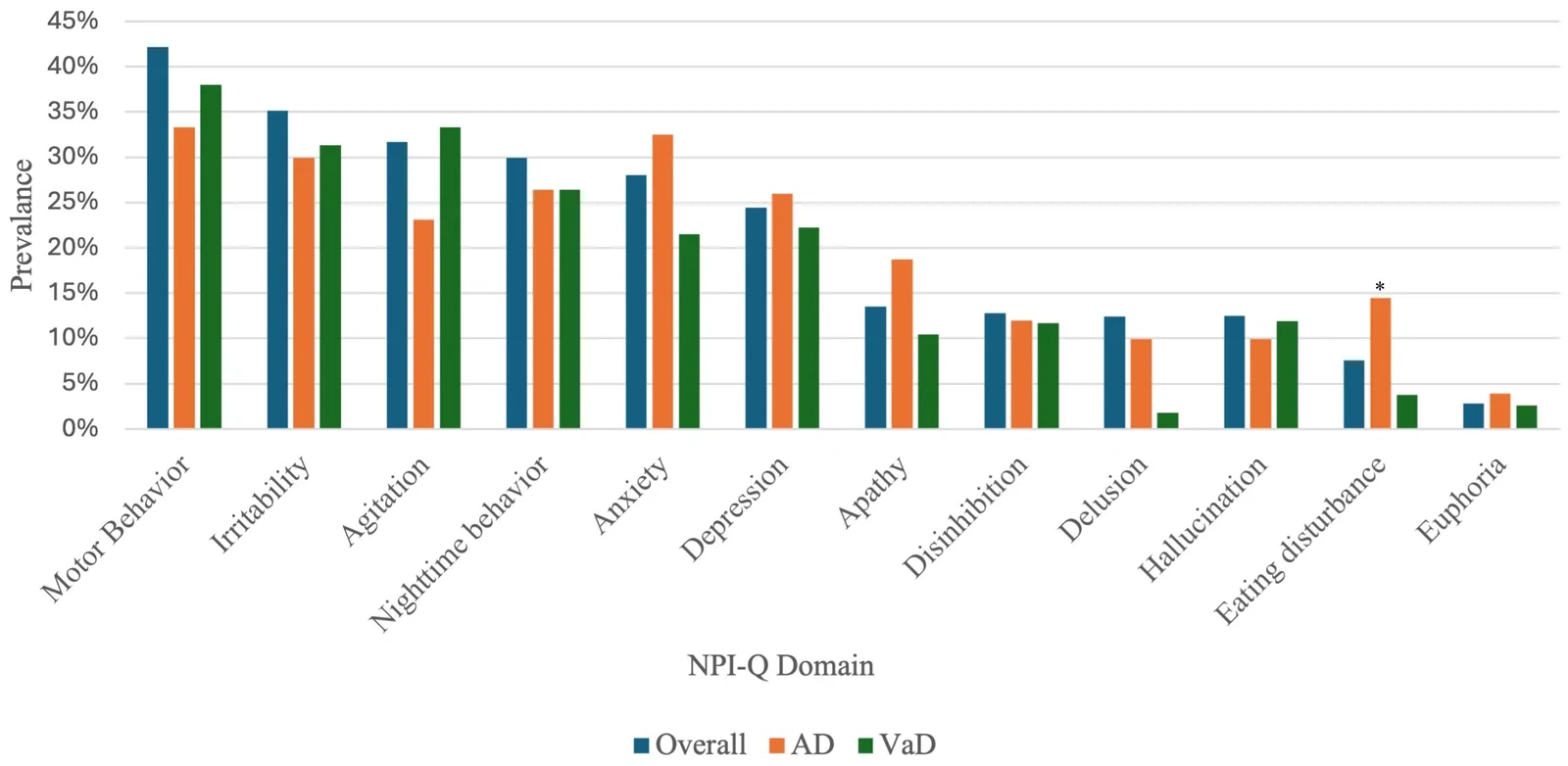
Comparison of neuropsychiatric symptom profiles across the total cohort, AD, and VaD groups. The grouped bar chart displays the prevalence (%) of each neuropsychiatric symptom based on the NPI-Q for overall. N varies by symptom due to missing responses (overall n range 243–267; AD 53–81; VaD 53–85). Symptoms are ordered by decreasing frequency within the overall group. Notably, AD patients exhibited a significantly higher prevalence of eating disturbances compared to the VaD group. *p < 0.05 denotes significant differences between AD and VaD groups, calculated using the Likelihood Ratio Chi-square test. AD, Alzheimer’s disease; VaD, vascular dementia; NPI-Q, Neuropsychiatric Inventory Questionnaire.

Overall, the median NPI-Q total severity score was low and did not differ significantly between AD and VaD (**Table 3**). TGDS scores also suggested comparable mild depressive symptoms between groups. Across the cohort, the most prevalent NPI-Q symptoms were motor behavior (42.2%), irritability/lability (35.1%), and agitation/aggression (31.7%). The distribution of individual neuropsychiatric symptoms across the overall cohort, AD, and VaD groups is shown in **Figure 3**. Eating disturbance showed a nominal unadjusted difference between AD and VaD, with a higher prevalence in AD (14.5% vs 3.8%, unadjusted p = 0.017). However, p-values for NPI-Q domain comparisons were adjusted for multiple testing using the Benjamini–Hochberg false discovery rate (q = 0.05), and no domain remained statistically significant after correction; therefore, domain-level findings should be interpreted as exploratory. Functionally, most patients remained independent in basic activities of daily living, while over one-third required assistance with instrumental activities; BADL and iADL status did not differ significantly between AD and VaD.

**Table 3:** Neuropsychiatric symptoms in young-onset dementia patients, overall and by dementia subtype (AD and VaD).

Neuropsychiatric symptoms	Overall*	AD	VaD	p-value†	BH-FDR q-value‡
Neuropsychiatric symptoms					
TGDS	6.57 ± 3.48 (n = 47)	5.86 ± 2.17 (n = 22)	7.36 ± 3.90 (n = 11)	0.164	–
NPI-Q total severity score, median (IQR)	1 (0, 3) (n = 180)	2 (0.25, 3.75) (n = 48)	2 (0, 6) (n = 59)	0.970	–
NPI-Q domain prevalence, n (%) - Motor behavior - Irritability/lability - Agitation/aggression - Nighttime behavior - Anxiety - Dysphoria/Depression - Apathy/Indifference - Disinhibition - Delusion - Hallucination - Eating disturbance - Euphoria	108 (42.2) (n = 256) 91 (35.1) (n = 259) 82 (31.7) (n = 259) 49 (29.9) (n = 164) 70 (28) (n = 250) 62 (24.4) (n = 254) 33 (13.5) (n = 250) 31 (12.8) (n = 243) 33 (12.4) (n = 266) 32 (12) (n = 267) 19 (7.6) (n = 254) 7 (2.8) (n = 248)	26 (33.3) (n = 78) 23 (29.9) (n = 77) 16 (23.1) (n = 75) 14 (26.4) (n = 53) 25 (32.5) (n = 77) 20 (26) (n = 77) 14 (18.7) (n = 75) 9 (12) (n = 75) 8 (9.9) (n = 81) 7 (8.8) (n = 80) 11 (14.5) (n = 76) 3 (3.9) (n = 76)	30 (38) (n = 79) 26 (31.3) (n = 83) 27 (33.3) (n = 81) 14 (26.4) (n = 53) 17 (21.5) (n = 79) 18 (22.2) (n = 81) 8 (10.4) (n = 77) 9 (11.7) (n = 77) 10 (11.9) (n = 84) 5 (5.9) (n = 85) 3 (3.8) (n = 79) 2 (2.6) (n = 75)	0.544 0.842 0.092 0.365 0.12 0.581 0.145 0.953 0.676 0.478 0.017 0.627	0.723 0.953 0.480 0.723 0.480 0.723 0.480 0.953 0.812 0.717 0.204 0.752
Function					
BADL - Independence - Partial dependence - Dependence iADL - Independence - Partial dependence - Dependence	(n = 189) 157 (83.1) 16 (8.5) 16 (8.5) (n = 182) 98 (53.8) 66 (36.3) 18 (9.9)	(n = 59) 50 (84.7) 4 (6.8) 5 (8.5) (n = 57) 33 (57.9) 17 (29.8) 7 (12.3)	(n = 52) 46 (88.5) 3 (5.8) 3 (5.8) (n = 51) 25 (49) 22 (43.1) 4 (7.8)	0.830 0.325	

Data are presented as n (%), mean ± SD, or median (interquartile range).

*Percentages and p-values for all columns (Overall, AD, and VaD) are calculated based on valid cases only (the number of patients with available data). The specific number of valid cases (n) for each analysis is provided in parentheses.

†Unadjusted p-values were calculated using the Likelihood Ratio Chi-square test for each NPI-Q domain prevalence, the Mann–Whitney U test for NPI-Q total severity score, and the independent samples t-test for TGDS to compare AD and VaD groups. For NPI-Q domain-level comparisons, p-values were additionally adjusted for multiple testing using the Benjamini–Hochberg false discovery rate (q = 0.05); no domain remained statistically significant after correction.

AD, Alzheimer’s disease; VaD, vascular dementia; TGDS, Thai Geriatric Depression Scale; NPI-Q, Neuropsychiatric Inventory Questionnaire; IQR, interquartile range; BADL, Basic Activities of Daily Living; iADL, Instrumental Activities of Daily Living.

### 3.4 Potential association of cognitive and neuropsychiatric symptoms with neuroimaging features in VaD

To screen for infectious serology and modifiable cardiometabolic co-morbidities, as well as to explore metabolic variations across subtypes, **Table 4** summarizes serology and metabolic profiles, with available data varying by test (n = 35–182). All tested participants had non-reactive anti-HIV results (35/35, 100%). Syphilis screening was non-reactive in 117/121 participants with available data (96.7%). Reactive non-treponemal screens were low-titer/weakly reactive and were not supported by confirmatory TPHA testing, suggesting biological false-positive results. FBS and HbA1C did not differ significantly between AD and VaD. In contrast, HDL was higher in AD than VaD (62.74 ± 15.41 vs 50.03 ± 14.18 mg/dL, p < 0.001), and LDL was also higher in AD (median 124 [IQR 82.5–147.5] vs 99.5 [66–142.25] mg/dL, p = 0.046). To partially address confounding, we additionally performed multivariable linear regression adjusting for age, sex, and education. With dementia subtype coded as AD = 1 and VaD = 2, subtype remained associated with HDL and LDL: VaD was associated with lower HDL compared with AD (B = −11.17 mg/dL, p = 0.007; 95% CI −19.22 to −3.12) and lower LDL compared with AD (B = −28.29 mg/dL, p = 0.039; 95% CI −55.07 to −1.52), while FBS and HbA1c remained non-significant. These findings should be interpreted cautiously given missing medication data (e.g., statin use).

**Table 4:** Unadjusted comparisons of the laboratory findings.

Lab parameters	Overall	AD	VaD	p-value†
Metabolic profiles				
FBS	99 (90, 115) (n = 182)	99 (89, 115) (n = 51)	100.5 (91, 117.25) (n = 62)	0.593
HbA1C	6.02 (5.6, 6.66) (n = 85)	5.9 (5.502, 6.77) (n = 22)	6.16 (5.6, 6.73) (n = 41)	0.724
HDL	55.168 ± 15.59 (n = 149)	62.739 ± 15.41 (n = 46)	50.03 ± 14.18 (n = 56)	< 0.001
LDL	110 (78, 148) (n = 155)	124 (82.5, 147.5) (n = 49)	99.5 (66, 142.25) (n = 58)	0.046

Laboratory findings in young-onset dementia patients, overall and by dementia subtype (AD and VaD).

Data are presented as n (%) for categorical variables, or as mean ± SD or median (interquartile range) as appropriate. n (valid) indicates the number of participants with available data.

†p-value compares AD and VaD groups only, calculated using Mann-Whitney U test or Independent T-test.

AD, Alzheimer’s disease; VaD, vascular dementia; HIV, Human Immunodeficiency Virus; FBS, fasting blood sugar; HbA1C, glycated hemoglobin; HDL, high-density lipoprotein; LDL, low-density lipoprotein; SD, standard deviation.

Neuroimaging characteristics among vascular dementia patients with available neuroimaging documentation are summarized in **Table 5** (denominators vary by imaging variable due to incomplete reporting). Small vessel disease (white matter hyperintensities and/or lacunar infarction) was the most frequent lesion type, and vascular lesions commonly involved cortical regions and the basal ganglia/internal capsule. Radiology-reported brain atrophy was observed in approximately one-quarter of VaD cases.

**Table 5:** Neuroimaging features of VaD.

Neuroimaging	Frequency
Lesion type, n (%)	
- Large vessel (Territorial infarction) - Small vessel (White matter hyperintensities/Lacunar infarction) - Cerebral hemorrhage	17 (23) (n = 74) 57 (77) (n = 74) 16 (21.9) (n = 73)
Topography, n (%)	
- Cortical area - Basal ganglion/internal capsule - Thalamus - Brainstem/cerebellum	33 (45.8) (n =72) 29 (39.2) (n = 74) 9 (12.2) (n = 74) 21 (28.4) (n = 74)
Brain atrophy, n (%)	
Atrophy	20 (26.7) (n = 75)

Neuroimaging and vascular characteristics of patients with vascular dementia.

Data are presented as n (%) using valid cases for each parameter; denominators are shown in parentheses. Percentages may exceed 100% within lesion-type and topography categories because multiple lesion types/topographies could be present in the same patient.

### 3.5 Clinical characteristics of bvFTD

Young-onset dementia in this study was defined as symptom onset before age 65 years. Within the FTD subgroup, diagnostic classification was performed using the FTDC criteria (13). The mean age at symptom onset was 54.45 ± 8.88 years, the median onset-to-diagnosis interval was 13 months (IQR 12–24), and females comprised 54.5% of cases. Behavioral change was the most frequent presenting complaint, and the most common FTDC features were executive-predominant deficits (90.9%), early loss of sympathy/empathy (63.6%), and early apathy/inertia (54.5%). Based on clinical features and supportive neuroimaging, 81.8% met criteria for probable bvFTD and 18.2% for possible bvFTD (**Table 6**).

**Table 6:** Clinical characteristics of bvFTD.

Characteristics	Frequency (n = 11)
Demographics	
Age at diagnosis (years), mean ± SD Age at onset (years), mean ± SD Symptoms onset-to-diagnosis (months), median (IQR) Sex: Female, n (%)	59.73 ± 7.96 54.45 ± 8.88 13 (12, 24) 6 (54.5)
Presenting Chief Complaints, n (%)	
- Memory impairment - Behavioral changes - Language problems - Motor or gait disturbance	5 (45.5) 7 (63.6) 1 (9.1) 1 (9.1)
Core FTDC Clinical Features, n (%)	
- Early behavioral disinhibition - Early apathy or inertia - Early loss of sympathy or empathy - Early perseverative, stereotyped or compulsive/ritualistic behavior - Hyperorality and dietary changes - Neuropsychological profile: executive/generation deficits with relative sparing of memory and visuospatial functions	4 (36.4) 6 (54.5) 7 (63.6) 5 (45.5) 0 (100) 10 (90.9)
Diagnosis (Rascovsky Criteria), n (%)	
- Possible bvFTD - Probable bvFTD	2 (18.2) 9 (81.8)

Clinical, demographic, and diagnostic characteristics of patients with behavioral variant frontotemporal dementia (bvFTD).

Data are presented as n (%) for categorical variables, mean ± SD or median (interquartile range) as appropriate.

*Percentages within the Presenting Chief Complaints and Core FTDC Clinical Features categories may not sum to 100% as individual patients could present with multiple overlapping clinical signs or symptoms at the time of evaluation.

bvFTD, behavioral variant frontotemporal dementia; FTDC, International Behavioral Variant FTD Criteria; IQR, interquartile range; SD, standard deviation.

## 4 Discussion

This retrospective cross-sectional study describes the clinical and neuropsychiatric profiles of young-onset dementia (YOD) in a tertiary-care cohort from Northern Thailand. Alzheimer’s disease and vascular dementia were the two most common subtypes in our sample, with a relatively high proportion of vascular dementia compared with many prior Asian clinic-based reports. Notably, brief cognitive screening measures (e.g., MSET-10/MoCA-based instruments) did not statistically differentiate Alzheimer’s disease from vascular dementia. However, differences may become clinically apparent when individual cognitive domains are analyzed; specifically, more detailed assessment of delayed recall and cueing effects, and orientation scores may provide diagnostic profiles for distinguishing between these conditions, as documented in previous study (14). Despite meaningful differences in presenting complaints, memory impairment predominating in Alzheimer’s disease and behavioral change appearing more frequently in vascular dementia. Behavioral and psychological symptoms were highly prevalent, particularly aberrant motor behavior and irritability, occurring alongside a substantial burden of subcortical and basal ganglia lesions on neuroimaging; together, these findings emphasize the need for earlier referral and multimodal assessment (detailed history, neuropsychological testing, and imaging) and support a more rational, risk-based approach to laboratory screening in contemporary YOD evaluations.

### 4.1 Age at onset and diagnostic delay

The average age at symptom onset and diagnosis in this cohort aligned with typical young-onset dementia patterns. Notably, the duration from symptom onset to diagnosis was approximately two years. This interval is considerably shorter than the global average, which typically ranges from 3.4 to 4.4 years (5, 6). These comparisons should be interpreted cautiously given differences in healthcare systems, study definitions of symptom onset and diagnosis, and case-mix across studies.

Several factors may contribute to the shorter diagnostic interval observed in this study, including close family observation in multigenerational households that may facilitate earlier recognition of subtle cognitive or behavioral change. However, this finding may also reflect referral pathways and selection toward more severe, atypical, or complex presentations that are more likely to reach a tertiary-care university hospital (15).

Access pathways within the Thai healthcare system may also influence time to specialist assessment (16). Although shorter than global report (5), a mean 2-year delay still represents a clinically important diagnostic gap. During this interval, patients may experience social health disruptions that lead to severe social frailty and isolation. Because symptom onset occurs during their active working years, these patients frequently experience abrupt employment loss, financial insecurity, and sudden shifts in family roles (17). Furthermore, delayed diagnosis significantly compromises optimal therapeutic windows. Crucially, it may cause patients to miss the eligibility timeline for initiating novel disease-modifying therapies (DMTs) for biologically proven Alzheimer’s disease, which are strictly restricted to patients in the mild cognitive impairment (MCI) or mild dementia stages (18). Reducing this delay should be a key service priority.

### 4.2 High prevalence of VaD and vascular topography

Globally, the age-standardized prevalence of young-onset dementia is 41.1 per 100,000 population for AD and 14.9 per 100,000 population for VaD (2). When scoping into Asia, regional clinical studies consistently highlight AD as the most common subtype. For example, in a Southeast Asian cohort from Singapore, AD accounts for 43.5% of young-onset dementia cases, followed by VaD at 18.1% (19). Similarly, in East Asia, clinic data from Hong Kong reveals that AD remains the leading cause, representing 70% of early-onset primary dementia cases (20). This trend is also consistent in Thailand, a multicenter registry study demonstrated that YOD represents 17% of all dementia patients, with early-onset amnestic AD being the leading cause (44%), followed by VaD (16%) (7). However, AD and VaD were observed at similar frequencies [AD (30.8%) and VaD (30.5%)], with FTD accounting for only 3.7% of cases in our study. Because this is a retrospective tertiary-care cohort, subtype proportions may not reflect community prevalence. In addition, mixed pathology may occur in YOD, and limited biomarker confirmation may have led to under-recognition of mixed AD/VaD cases, as well as other atypical proteinopathies and neurodegenerative conditions that mimic or overlap with these common subtypes, such as corticobasal degeneration, dementia with Lewy bodies, or frontotemporal dementia associated with motor neuron disease. The relatively high proportion of VaD observed in our study appears to be closely linked to the significant burden of established vascular risk factors within our cohort, including hypertension, dyslipidemia and prior stroke or TIA. These findings align with epidemiological data in Thailand, the study found that the age-standardized prevalence of metabolic syndrome by International Diabetes Federation (IDF) and National Cholesterol Education Program III (NCEP) criteria were 24.0% and 32.6%, respectively (21).

This metabolic burden directly aligns with the observed vascular topography in our study, where subcortical small vessel pathology predominated, primarily clustering within the basal ganglia and internal capsule. From a neurovascular perspective, chronic cardiometabolic risk factors originating in early adulthood accelerate arteriolosclerosis, atherosclerosis, and microvascular disease (22). This pathomechanism damages fragile cerebral perforating vessels, particularly the lenticulostriate arteries, which are vulnerable to chronic high blood pressure. The resulting vascular injury drives the high rate of deep subcortical lesions—namely lacunar infarcts and white matter hyperintensities—predominating within our cohort. The structural disruption in these deep brain regions may disrupt frontal-subcortical circuits, potentially contributing to a disconnection syndrome that impairs cognitive network efficiency (23). This framework is hypothesized to underlie the observed association with young-onset VaD cognitive profiles are characterized by prominent, gradual deficits in processing speed, complex attention, and executive functions rather than isolated memory impairment (22), consistent with the notion that metabolic syndrome may contribute to target-organ brain injury in younger populations.

### 4.3 Clinical presentations and BPSD

YOD is known to present complex symptoms, frequently involving language difficulties, behavioral changes, and executive dysfunction rather than isolated memory loss (24). In our study, while memory impairment was the most frequent overall chief complaint (78.3%), behavioral changes were also notably prominent (29.2%). When stratified by subtype, memory impairment was characteristically dominant in the AD group, whereas VaD patients exhibited higher rates of behavioral symptoms. This is consistent with prior report showing predominant memory symptoms in AD (25), vascular cognitive impairment is frequently accompanied by subcortical ischemic changes that manifest as executive dysfunction (22) and late-life depression (26). The predominance of non-amnestic symptoms, particularly behavioral changes in our vascular dementia group, aligns with recent neuroimaging evidence in the Thai population demonstrating that microstructural changes in white matter tracts are significantly associated with cognitive domains beyond episodic memory, such as attention and executive function, in individuals with non-amnestic impairment (27). This supports our finding that vascular-related damage to subcortical circuits likely manifests as executive and behavioral disturbances rather than the classic memory loss seen in Alzheimer’s disease.

Despite these clinical presentations, our cognitive screening instruments, MoCA, TMSE, and MSET-10, failed to statistically differentiate between AD and VaD. However, in sensitivity analyses incorporating education via multiple imputation, the MSET-10 AD–VaD contrast was borderline and the confidence interval crossed the null (pooled p = 0.053; 95% CI spanning 0), indicating that a modest subtype difference cannot be excluded; therefore, these negative findings should be interpreted cautiously given retrospective test selection and limited power in adjusted models. This finding is consistent with the known psychometric limitations of brief cognitive screening tools in dementia subtyping. The MSET-10, in particular, exhibits ceiling effects for many subitems and demonstrates limited sensitivity to frontal-executive and visuospatial impairments that are characteristic of vascular cognitive impairment (28). While the MoCA was designed to address some of these limitations by incorporating more challenging executive function and visuospatial tasks, systematic reviews have documented that both instruments have suboptimal psychometric properties in cerebrovascular disease populations (29). Furthermore, these screening instruments assess multiple cognitive domains in a time-limited format, which may result in insufficient domain-specific depth to capture the nuanced cognitive profiles (30). The inability of these tools to differentiate subtypes in our young-onset dementia cohort underscores the necessity of comprehensive neuropsychological assessment and neuroimaging for accurate diagnostic classification, rather than reliance on brief screening measures alone.

Regarding BPSD, our study identified motor disturbances as the most prevalent symptom, followed by a notably high prevalence of irritability. The prominence of irritability is consistent with international young-onset dementia cohorts, where irritability, agitation, and apathy are frequently reported as the most common neuropsychiatric symptoms (9, 31, 32). However, the high rate of aberrant motor behavior in our sample warrants particular attention. This finding likely reflects the high proportion of vascular dementia cases in our cohort and the characteristic localization of vascular lesions to the basal ganglia and subcortical white matter observed in our neuroimaging data. While lesion localization plausibly contributes to these neuropsychiatric manifestations, our cross-sectional design cannot establish causal relationships between imaging burden and BPSD severity. Basal ganglia structures, particularly the caudate nucleus and globus pallidus, are integral components of parallel frontal-subcortical circuits that mediate motor programming, behavioral initiation, and motivational processes (33). Ischemic lesions in these regions, as well as disruption of their white matter connections, have been associated with disconnection syndromes characterized by motor restlessness, perseverative behaviors, and parkinsonian features (34, 35). The high vascular dementia prevalence, basal ganglia lesion burden, and elevated motor behavior symptoms in our cohort demonstrate an observed co-occurrence that supports the hypothesized neuroanatomical association of BPSD profiles in young-onset dementia, highlighting the potential importance of structural lesion localization in predicting neuropsychiatric manifestations.

### 4.4 Characteristics of bvFTD

In the present cohort, behavioral variant frontotemporal dementia accounted for 3.7% (n=11) of all young-onset dementia cases, with a mean age at diagnosis of 59.73 ± 7.96 years and a median symptom onset-to-diagnosis interval of 13 months. Despite the small sample size limiting statistical power, applying the revised FTDC criteria yielded a high rate of probable bvFTD (81.8%). In our study, the most frequently observed core features were early loss of sympathy or empathy (63.6%), early apathy or inertia (54.5%), and early perseverative, stereotyped, or compulsive behaviors (45.5%), while hyperorality was notably absent. Importantly, 90.9% of patients exhibited the characteristic neuropsychological profile of executive and generation deficits with relative sparing of memory and visuospatial functions (36). The prominence of loss of empathy and sympathy in our cohort aligns with the recognized importance of social cognitive deficits in bvFTD, which map to frontomedian and insular network dysfunction and serve as key discriminating features driven by right-sided neurodegenerative changes within the anterior temporal lobe, medial orbitofrontal cortex, and anterior insula (36). These findings underscore the clinical utility of the FTDC framework in identifying bvFTD with high diagnostic certainty when combined with comprehensive neuropsychological assessment.

### 4.5 Laboratory findings and serology

In this study, routine serologic screening for infectious causes yielded uniformly negative results for HIV and predominantly negative results for syphilis. The small proportion of reactive syphilis serology consisted exclusively of low-titer or weakly reactive non-treponemal tests that were subsequently confirmed negative by treponemal-specific assays (TPHA). These findings align with contemporary evidence demonstrating that neurosyphilis and HIV-associated dementia have become uncommon contributors to young-onset dementia in the modern era of combination antiretroviral therapy and improved public health infrastructure (37). In the context of Thailand’s robust HIV prevention and effective syphilis control measures, the absence of confirmed infectious causes in our tertiary referral cohort likely reflects public health success (38, 39) and the changing epidemiology of young-onset dementia. Importantly, a comprehensive screening panel, including syphilis serology, thyroid function tests, liver function tests, and a complete blood count, remains appropriate in routine clinical practice to rule out treatable mimics or coexisting reversible causes of cognitive decline, particularly in settings where the incidence of these conditions remains substantial.

Our study found a discrepancy in lipid profiles between AD and VaD patients, with the VaD group paradoxically demonstrating healthier lipid levels compared to the AD group. One possible explanation is the intensive secondary prevention and vascular risk management applied to patients with vascular diseases (40). Because people with VaD typically have a prominent history of cerebrovascular accidents, such as stroke, they receive more aggressive lipid-lowering protocols initially, which may partly account for the observed differences. This treatment-related confounding underscores the importance of careful interpretation of cross-sectional lipid data in dementia cohorts and highlights the need for longitudinal studies that account for medication exposure when examining the relationship between lipid metabolism and dementia pathogenesis.

Importantly, data on concurrent statin prescriptions and other lipid-modifying medications were not available in this study, and no multivariable regression was performed to adjust for these potential confounders, as well as age and sex. The absence of medication data therefore represents a primary analytical limitation, and these univariate lipid findings should be interpreted with considerable caution rather than reflecting intrinsic metabolic differences between dementia subtypes.

### 4.6 Implications for practice

In young-onset dementia, careful history-taking and collateral information from informants are critical for early subtyping, particularly to differentiate Alzheimer’s disease from vascular dementia when presenting complaints diverging. Brief cognitive screening instruments alone appear insufficient for reliable subtype discrimination; clinicians should prioritize comprehensive neuropsychological assessment and multimodal evaluation with neuroimaging. Furthermore, where accessible, the incorporation of validated fluid or molecular biomarkers (e.g., cerebrospinal fluid or PET imaging) is increasingly important for enhancing etiological diagnostic certainty, providing precise prognostication, and guiding personalized care for PwD. Given the substantial vascular burden observed in this cohort, early identification and aggressive management of vascular risk factors should be integrated into YOD care pathways. Behavioral and psychological symptoms should be anticipated and managed proactively, especially irritability and aberrant motor behavior, using structured assessment and individualized nonpharmacologic strategies with judicious medication use when needed. Finally, in similar low-prevalence settings, infectious serology may be considered using a targeted, risk-based approach, while maintaining testing when clinical features or risk factors raise suspicion.

### 4.7 Limitations

First, this was a retrospective, single-center study conducted at a university hospital. Because we relied on historical medical records, some variables were incompletely documented, particularly detailed psychiatric descriptions and longitudinal cognitive trajectories. Second, the cohort is subject to referral (selection) bias inherent to tertiary-care settings, where people with young-onset dementia (YOD) may present with more complex, atypical, or severe conditions. Consequently, subtype distribution and symptom severity observed in this study may not be generalizable to patients managed in community or primary care settings. Third, the cross-sectional design provides only a single time-point assessment and precludes evaluation of disease progression, longitudinal functional outcomes, treatment response, and changes in caregiver burden over time.

Fourth, dementia subtyping was based on clinical criteria and routine neuroimaging (CT/MRI), reflecting real-world practice in resource-limited settings. However, the absence of etiology-specific biomarkers (e.g., CSF amyloid-beta/tau or amyloid PET) may have limited diagnostic precision, particularly for distinguishing Alzheimer’s disease from vascular dementia and for identifying mixed pathologies. Fifth, dementia diagnoses were extracted from ICD-10 codes in medical records; while practical for retrospective registry reviews, the lack of standardized application of newer domain-specific consensus criteria may have introduced diagnostic heterogeneity.

Sixth, missing data were substantial for several variables, and cognitive screening instruments were not uniformly administered. Test availability differed by instrument (MoCA n = 138, TMSE n = 104, and MSET-10 n = 156 overall), and subgroup comparisons between AD and VaD were therefore based on smaller instrument-specific samples (e.g., MSET-10: AD n = 50; VaD n = 51). Education was missing in 48.5% of participants. We assessed the missing-data mechanism using Little’s MCAR test (χ² = 1.515, df = 1, p = 0.218) for the variables included in the test and summarized missingness patterns; nevertheless, MCAR cannot be guaranteed for all study variables, and residual selection bias remains possible. To evaluate robustness to listwise deletion, we performed sensitivity analyses using multivariable regression within cognitive-test subsamples and repeated models including education using multiple imputation (m = 5). While these analyses supported the overall conclusions, some adjusted estimates (particularly MSET-10) were borderline and should be interpreted cautiously. In addition, non-significant differences in cognitive screening scores between AD and VaD should not be interpreted as evidence of equivalence, as modest between-group differences may not have been detectable for given instrument-specific sample sizes. Seventh, sample sizes for less common dementia subtypes (e.g., frontotemporal dementia) were small, limiting statistical power for subgroup analyses and restricting the strength of inference for these diagnoses. Eighth, medication data, including statin prescription and other lipid-lowering therapy, antihypertensive regimens, and antidiabetic treatment, were not systematically extracted. Consequently, metabolic and lipid comparisons may be confounded by treatment exposure and secondary prevention intensity. To partially address confounding, we performed multivariable linear regression for FBS, HbA1c, HDL, and LDL adjusting for age, sex, and education; however, because statin use and other relevant treatments were unmeasured, residual confounding remains, and causal interpretation of lipid differences between AD and VaD is not possible.

## 5 Conclusion

This retrospective study provides key insights into young-onset dementia (YOD) in Northern Thailand. Chief complaints differ significantly between dementia subtypes, with memory impairment predominating in Alzheimer’s disease and behavioral changes occurring more frequently in vascular dementia. However, brief cognitive screening tools did not statistically differentiate Alzheimer’s disease from vascular dementia, highlighting the need for detailed neuropsychological testing and advanced neuroimaging.

Motor-related behavioral symptoms were common in this cohort and occurred alongside a substantial burden of subcortical and basal ganglia lesions on neuroimaging, supporting the clinical relevance of vascular lesion topography when anticipating neuropsychiatric manifestations. Infectious serologic testing revealed no evidence of active HIV-associated dementia or neurosyphilis, which may support consideration of a more targeted, risk-based screening approach in similar settings while maintaining testing when clinical features or risk factors warrant. Future research should prioritize longitudinal, multicenter collaborative studies across Southeast Asia to track disease progression, validate culturally adapted diagnostic tools, and incorporate advanced biomarkers. Finally, prospective studies evaluating caregiver burden, quality of life, and healthcare utilization are essential to build better healthcare policies and support systems for YOD families in resource-limited settings.

## Data Availability

The original contributions presented in the study are included in the article/supplementary material. Further inquiries can be directed to the corresponding author.
